# Parents' acceptance to vaccinate children against COVID-19: A Syrian online survey

**DOI:** 10.3389/fpubh.2022.955362

**Published:** 2022-10-13

**Authors:** Sarya Swed, Hidar Alibrahim, Haidara Bohsas, Sheikh Shoib, Mohammad Mehedi Hasan, Karam R. Motawea, Mhd Kutaiba Albuni, Elias Battikh, Bisher Sawaf, Nashaat Kamal Hamdy Elkalagi, Safaa Mohamed Alsharief Ahmed, Eman Mohammed Sharif Ahmed, Lina Taha Khairy, Agyad Bakkour, Ali Hadi Hussein Muwaili, Fatima Abubaker Abdalla Abdelmajid, Dhuha Hadi Hussein Muwaili, Mohamed Elsayed, Shoaib Ahmad, Ka Yiu Lee

**Affiliations:** ^1^Faculty of Medicine, Aleppo University, Aleppo, Syria; ^2^Department of Psychiatry, Jawahar Lal Nehru Memorial Hospital, Srinagar, Kashmir, India; ^3^Department of Biochemistry and Molecular Biology, Faculty of Life Science, Mawlana Bhashani Science and Technology University, Tangail, Bangladesh; ^4^Faculty of Medicine, Alexandria University, Alexandria, Egypt; ^5^Department of Internal Medicine, Damascus University, Damascus, Syria; ^6^Department of Internal Medicine, Syrian Private University, Damascus, Syria; ^7^Lecturer in Internal Medicine and Tropical Medicine at Faculty of Medicine Al Arish University, Al Arish, Egypt; ^8^Faculty of Medicine, Shendi University, Shendi, Sudan; ^9^Faculty of Medicine, Nile Valley University, Atbara, Sudan; ^10^Faculty of Medicine, The National Ribat University, Al Khurtum, Sudan; ^11^Faculty of Medicine, Albaath University, Homs, Syria; ^12^Ivano-Frankivsk National Medical University, Ivano-Frankivsk Oblas, Ukraine; ^13^Faculty of Medicine, University of Medical Sciences and Technology, Al Khurtum, Sudan; ^14^Department of Psychiatry and Psychotherapy III, University of Ulm, Ulm, Germany; ^15^Department of Psychiatry, School of Medicine and Health Sciences, Carl von Ossietzky University Oldenburg, Oldenburg, Germany; ^16^Swedish Winter Sports Research Centre, Department of Health Sciences, Mid Sweden University, Sundsvall, Sweden

**Keywords:** COVID-19, vaccine, children, Syria, parents

## Abstract

After the widespread of COVID-19 virus worldwide, vaccination targeted reducing spread of cases and mortality rates. However, vaccination hesitancy was observed among the communities worldwide. Vaccination hesitancy involved parents regarding the decision of vaccinating their children- After obtaining ethical approval, an online cross-sectional study was conducted from 1 March to 22 April 2021 to evaluate the parents' acceptance of vaccinating their children against the COVID-19 virus in Syria. Data were analyzed using descriptive and multivariate logistic regression analysis in IBM, SPSS V. 28.0 package program (IBM Corporation, Armonk, NY, USA). Among 283 participants, 105 participants agreed to vaccinate their children, and 178 were not. A significant correlation between age and vaccine willingness was found (*P*-value < 0.0001^*^), especially in the age group between 18 and 30 years old (45.2%). Parents who accepted vaccinating themselves were more willing to vaccinate their children (34.6%). According to our results, there is a greater need to enhance awareness and knowledge programs about the vaccine's effectiveness and encourage parents to accept giving the vaccine to their children.

## Background

According to WHO, until 20 May 2021, there were over 160 million confirmed cases of COVID-19 worldwide and more than three million deaths (1). Developing an efficient and safe vaccination for all populations, including children, is an effective strategy for reducing COVID-19 death rates and increasing immunity levels in society (2). Many local and international institutions have attempted to develop many vaccines since the COVID-19 pandemic. In March 2021, the efficacy of 44 COVID-19 vaccines was evaluated by randomized clinical trials; another 151 vaccines are within preclinical testing to estimate their efficacy and safety for COVID19 patients (3). The world health organization verified several COVID-19 vaccines' efficacy and safety, where the first vaccination program started in early December 2020, and the number of vaccination doses is updated daily. According to the Syrian Ministry of Health, the authorized number of confirmed cases in Syria is, to the date of writing, 24,700 cases (4). However, the current reports of confirmed cases are not precise due to the lack of conducted tests to confirm the diagnosis of COVID-19 and their relatively expensive cost. In addition, the Syrian population has faced the war for 10 years; thus, the COVID-19 pandemic has increased the health, social, and educational crises and led to creating a double crisis (5). Global efforts for vaccination are assumed to protect up to 2–3 million lives by priming the immune system against infections that threaten world health (6). No clear guideline or strategy have been adopted by Syrian health authorities regarding children COVID-19 vaccinations. Vaccine hesitancy was considered one of the top ten challenges to world health by the World Health Organization (WHO) in 2019 (4). Vaccination acceptance in societies depends on several factors such as education, income, and awareness levels (7). Trust in the health authorities, believing that the vaccine is dangerous or even useless, and unverified sources of information, may all be additional factors to affect vaccination acceptance rates. Several studies have found that vaccination acceptance is related to the individual's age. Older adults were more likely to accept the vaccine; on the other hand, individuals with higher incomes were more likely to receive the vaccine (7). Recipients of vaccines probably tend to reject the authenticity of lesser-known and local manufacturers, which might explain the lower vaccine acceptance rate (8). The efforts for vaccine acceptance should discourse society's fears, ignorance, historical factors that engender distrust, and religious or philosophical convictions (9). Physician advice is an important factor that might affect acceptance of vaccination against COVID-19 among residents (10). A cross-sectional study conducted in Syria on 7,531 individuals revealed that only 37% of the participants were accepting the vaccination if it was available, and 31% were uncertain (11). Nevertheless, many factors can influence parents' support of the new COVID-19 immunization schedule, including concerns about the vaccine safety or potential adverse events (12). To the best of our knowledge, the current literature lacks studies conducted in Syria on parents' willingness to vaccinate their children against COVID-19. This study aims to assess parental acceptance of enrolling their children in a COVID-19 vaccination program.

## Methods

### Study design and participants

This cross-sectional study was conducted in Syria from 1 March to 22 April 2021 to evaluate the parents' willingness and attitudes to vaccinate children against COVID-19. The ethical approval of the study was obtained from the Ethics Committee at Aleppo and Damascus University. The questionnaire was taken from a previous study conducted in Saudi Arabia to assess parents' willingness to vaccinate their children under 18 years of age. We translated the questionnaire from English to Arabic and made it suitable for Syrian parents' accents (12). Inclusion criteria were voluntary participation, parents of a child under 18, and living in Syria. Parents received all necessary information about the study, including the study's objectives, their right to withdraw from the study, information about confidentiality, and data protection, as well as the fact that only fully registered data would be considered for data analysis. We saved the data by a safe method, in which the responses were kept on the Google form website and then transferred to an excel sheet when finishing data collection. After agreeing to participate in the study, the parents gave their consent online. We conducted an experimental survey on 30 participants before distributing the questionnaire to test the usability and technical performance of the online survey. Furthermore, e tested the survey validity using a pilot version for all items completed by 25 parents from the target population who provided feedback regarding the comprehensibility of the questions and the time to complete the survey. Those 25 parents were not part of the actual survey. The tool's internal consistency of the used questionnaire was shown by Cronbach's alpha values, ranging from 0.7 to 0.8. Inclusion criteria were voluntary participation, parents of a child under the age of 18, and living in Syria. According to inclusion criteria, only 283 parents from 298 participants (participation rate = 95%) were eligible to be included in the present study. We created an online questionnaire using a Google form. The invitation messages were sent along with the questionnaire link to the participants through social media, including Facebook, WhatsApp, and telegram. The participants also had the option of sharing the survey link. Only Arabic versions of the survey tool were available. A single population proportion formula [*n* = [(Za/2)2. P (1–P)]/d2] was used to calculate the necessary sample size. Assuming a 95% confidence level (Z a/2 = 1.96), a 6% margin of error, and *P* = percentage of parents in Saudi Arabia that refuse to vaccinate their children (44 %). The required sample size was raised to 263. Six respondents refused to take part in the research, leaving us with 283 survey responses as the total sample size.

The survey includes questions about the parents' opinions and willingness to vaccinate their children under 18 with the COVID-19 vaccine, COVID-19 vaccination availability, reasons of willing vaccination.

The questionnaire was uploaded as Supplementary material.

### Statistical analysis

The Statistical Package for the Social Sciences (IBM SPSS V. 28.0) application was used to analyze the data. For statistical significance, a p-value of less than 0.05 was considered. The descriptive data of sociodemographic characteristics were shown as frequencies and percentages. By using the Mann-Whitney U-test (for non-normal continuous variables), t-test (for normal distribution of continuous variables), and chi-squared test, we conducted univariate analysis to identify parental characteristics that were related to their willingness to vaccinate children with a COVID-19 vaccine (for categorical variables). For the statistically significant results (*p* 0.05) in the univariate analysis, the odds ratios of receiving a COVID-19 vaccination for kids were next assessed using multivariate logistic regression analysis.

## Results

### Demographic characteristics of the study sample

We acquired 298 responses to the survey, six declined to participate in the study, and 283 was the final number of the study sample. The majority of participants were aged between 18 and 30(63.3%), composed of 138 females (48.8%) and 145 males (51.2%), 280 of them were Syrian citizens (98.9%), married (74.9%), with more than four family members (74.9%), moderate socioeconomic status (46.6%), most of them were occupied in the governmental sector (36.7%). Furthermore, most of the participants were postgraduate and were not working in a healthcare system (68.9%) (Table 1).

**Table 1:** Sociodemographic characteristics of the parents assessed for their willingness to vaccinate their children with a COVID-19 vaccine.

**Variables**	**Categories**	**Frequency (%)**
Age	18–30	179 (63.3%)
	31–40	34 (12%)
	>40	70 (24.7%)
Gender	Female	138 (48.8%)
	Male	145 (51.2%)
Nationality	Syrian	290 (98.9%)
	Non-Syrian	3 (1.1%)
Marital status	Married	212 (74.9%)
	Separated	61 (21.6%)
	Widower	7 (2.5%)
	Divorced	3 (1.1%)
Family members	1	7 (2.5%)
	2	8 (2.8%)
	3	28 (9.9%)
	4	66 (23.3%)
	>4	174 (61.5%)
Socioeconomic status	Low	33 (11.7%)
	Moderate	132 (46.6%)
	Good	98 (34.6%)
	High	20 (7.1%)
Occupation	Not working	94 (33.2%)
	Self-employed	39 (13.8%)
	Private sector	46 (16.3%)
	Governmental sector	104 (36.7%)
Education	High school or below	50 (17.7%)
	Illiterate	9 (3.2%)
	Pre-Graduate	117 (41.3%)
	Post-graduate	107 (37.8%)
Working in a healthcare system	Yes	88 (31.1%)
	No	195 (68.9%)

### Differences in parents' willingness to vaccinate their children

Our results have shown that 105 (37.1%) of the participants were willing to vaccinate their children, whereas 178 (62.9%) refused to vaccinate them. There was a significant correlation between the age and the willingness to vaccinate the child, specifically the age group 18 to 30, compared to other age groups. Despite this, no significant accusations were found between other variables and the child's willingness to vaccinate (Table 2).

**Table 2:** Parental characteristics associated with the willingness to vaccinate their children with a COVID-19 vaccine.

**Variables**	**Categories**	**Willing to vaccinate the child**	**Not willing to vaccinate the child**	***P*** **-value**
		105 (37.1%)	178 (62.9%)	
Age	18–30	51 (18%)	128 (45.2%)	<0.0001^*^
	31–40	18 (6.4%)	16 (5.7%)	
	>40	36 (12.7%)	34 (12%)	
Gender	Female	47 (16.6%)	91 (32.2%)	0.3
	Male	58 (20.5%)	87 (30.7%)	
Nationality	Syrian	105 (37.2%)	175 (61.8%)	0.18
	Non-Syrian	0 (0.0%)	3 (1.1%)	
Marital status	Married	85 (30%)	127 (44.9%)	0.295
	Separated	16 (5.7%)	45 (15.9%)	
	Widower	3 (2.9%)	4 (1.4%)	
	Divorced	1 (0.4%)	2 (0.7%)	
Family members	1	60 (21.2%)	114 (40.3%)	0.135
	2	5 (1.8%)	2 (0.7%)	
	3	4 (1.4%)	4 (1.4%)	
	4	14 (4.9%)	14 (4.9%)	
	>4	22 (13.3%)	44 (15.5%)	
Socioeconomic status	Low	16 (5.7%)	17 (6%)	0.257
	Moderate	46 (16.3%)	86 (30.4%)	
	Good	33 (11.7%)	65 (23%)	
	High	10 (3.5%)	10 (3.5%)	
Occupation	Not working	29 (10.2%)	65 (23%)	0.067
	Self-employed	13 (4.6%)	26 (9.2%)	
	Private sector	14 (4.9%)	32 (11.3%)	
	Governmental sector	49 (17.3%)	55 (19.4%)	
Education	High school or below	19 (6.7%)	31 (11%)	0.239
	Illiterate	3 (1.1%)	6 (2.1%)	
	Pre-Graduate	36 (12.7%)	81 (28.6%)	
	Graduated	47 (16.6%)	60 (21.2%)	
Working in a healthcare system	Yes	34 (12%)	54 (19.1%)	0.72
	No	71 (25.1%)	124 (43.8%)	

* *P*-value < 0.05.

However, parents who did not accept to participate in the COVID-19 vaccine clinical trial (21.9%) were more willing to vaccinate their children (*P* < 0.0001). Furthermore, parents who trust the healthcare system (29%) were more willing to vaccinate their children (*P* < 0.013). In contrast, parents with less confidence in domestic vaccines (25.1%) were more willing to vaccinate their children (*P* < 0.003). Approximately there was a significant correlation between parents that were concerned about being infected or someone in their family was infected with the COVID-19 virus (31.8%) and willingness to vaccinate their children (*P* < 0.037). Parents who did not refuse to vaccinate themselves or child because they considered it useless or dangerous were more willing to vaccinate their children (*P* < 0.0023). However, parents who did not postpone a vaccine for themselves or a child recommended by a physician (30.4%) were more willing to vaccinate their children (*P* < 0.037). The results show that parents who had not vaccinated themselves or a child despite doubts about its efficacy (21.9%) were more willing to vaccinate their children (*P* < 0.0001). However, parents who did not receive a seasonal flu vaccine (19.4%) were more willing to vaccinate their children (*P* < 0.0001).

Table 3 demonstrate that parents who agreed to vaccinate themselves (34.6%) with the COVID-19 vaccine were more willing to vaccinate their children (*P* < 0.0001).

**Table 3:** Parents' attitudes and opinions associated with their willingness to vaccinate their children with a COVID-19 vaccine.

**Parents' attitudes and opinions associated with a covid-19 vaccine administration to their children**	**Willing to vaccinate the child**	**Not willing to** **vaccinate the child**	**Total**	***P*** **-value**
	105 (37.1%)	178 (62.9%)	283	
Willingness to vaccinate themselves with a COVID-19 vaccine				<0.001^*^
No	7 (2.5%)	120 (42.4%)	127 (44.9%)	
Yes	98 (34.6%)	58 (20.5%)	156 (55.1%)	
Willingness to participate in a COVID-19 vaccine clinical trial				<0.001^*^
No	62 (21.9%)	161 (56.9%)	223 (78.8%)	
Yes	43 (15.2%)	17 (6%)	60 (21.2%)	
Trust in the healthcare system				0.013^*^
No	23 (8.1%)	64 (36%)	87 (30.7%)	
Yes	82 (29%)	114 (40.3%)	196 (69.3%)	
Confidence in domestic vaccines				0.003^*^
No	71 (25.1%)	148 (52.3%)	219 (77.4%)	
Yes	34 (12%)	30 (10.6%)	64 (22.6%)	
Concerned about being infected or someone in their family with the COVID-19 virus				0.037^*^
No	15 (5.3%)	44 (15.5%)	59 (20.8%)	
Yes	90 (31.8%)	134 (47.3%)	224 (79.2%)	
Refused a vaccine for themselves or a child because they considered it useless or dangerous				0.0023^*^
No	89 (31.4%)	130 (45.9%)	219 (77.4%)	
Yes	16 (15.2%)	48 (17%)	64 (22.6%)	
Postponed a vaccine for themselves or a child, recommended by a physician				0.037^*^
No	86 (30.4%)	126 (44.5%)	212 (74.9%)	
Yes	19 (18.1%)	52 (18.4%)	71 (25.1%)	
Had a vaccine for themselves or a child despite doubts about its efficacy				<0.001^*^
No	62 (21.9%)	146 (51.6%)	208 (73.5%)	
Yes	43 (15.2%)	32 (11.3%)	75 (26.5%)	
Received a seasonal flu vaccine				<0.001^*^
No	55 (19.4%)	136 (48.1%)	191 (67.5%)	
Yes	50 (17.7%)	42 (14.8%)	92 (32.5%)	

* *P*-value < 0.05.

The logistic regression model was statistically significant, X2(21) = 33.14, *p*-value=0.045. Hosmer and Lemeshow test: eight, The model explained 15.1% (Nagelkerke R Square) of the variance in Parents' Willingness to Vaccinate Children against COVID-19. Of seven predictor variables for willing patients to vaccinate the children, only the age variable has a statistically significant association (*P*-value = 0.012^*^). The parents under 30 years have a lower 0.34 times willingness than the parents above 40 years; thus, the acceptance of children's vaccination increased with elder age (Table 4).

**Table 4:** Predictors related to the parents' acceptance to vaccinate their children with a COVID-19 vaccine.

**Variable**	**Categories**	**OR**	**95%CI for B**		***P*** **-value**
			**Lower**	**Upper**	
Age					0.012^*^
	18–30	0.34	0.16	0.7	0.007^*^
	31–40	1.011	0.38	2.64	0.98
	>40	1	
Gender	Female	0.75	0.43	1.3	0.318
	Male	1	
Nationality	Syrian	907,699,260	000	-	0.999
	Non-Syrian	1	
Marital status					0.91
	Married	1.1	0.078	14.48	0.96
	separated	0.86	0.06	12.42	0.91
	Widower	1.4	0.068	32.22	0.80
	Divorced	1	
Family members					0.27
	1	4.7	0.7	29.9	0.1
	2	1.6	0.34	7.7	0.53
	3	1.9	0.79	5.1	0.156
	4	0.9	0.47	1.8	0.82
	>4	1	
Socioeconomic Status					0.35
	Low	1.29	0.33	4.9	0.71
	Medium	0.66	0.21	2.09	0.48
	Good	0.64	0.20	2.04	0.45
	High	1	
Occupation					0.53
	Not working	0.86	0.42	1.7	0.68
	Self-employed	0.81	0.33	1.9	0.63
	Private sector	0.53	0.23	1.23	0.13
	Governmental sector	1	
Education					0.81
	Illiterate	0.51	0.1	2.46	0.40
	High school or below	0.91	0.4	2.05	0.81
	Pre-graduate	1.1	0.53	2.42	0.73
	Graduate	1	
Working in a healthcare system	Yes	0.85	0.46	1.6	0.623
	No	1	

* *P*-value < 0.05.

**Figure 1:**
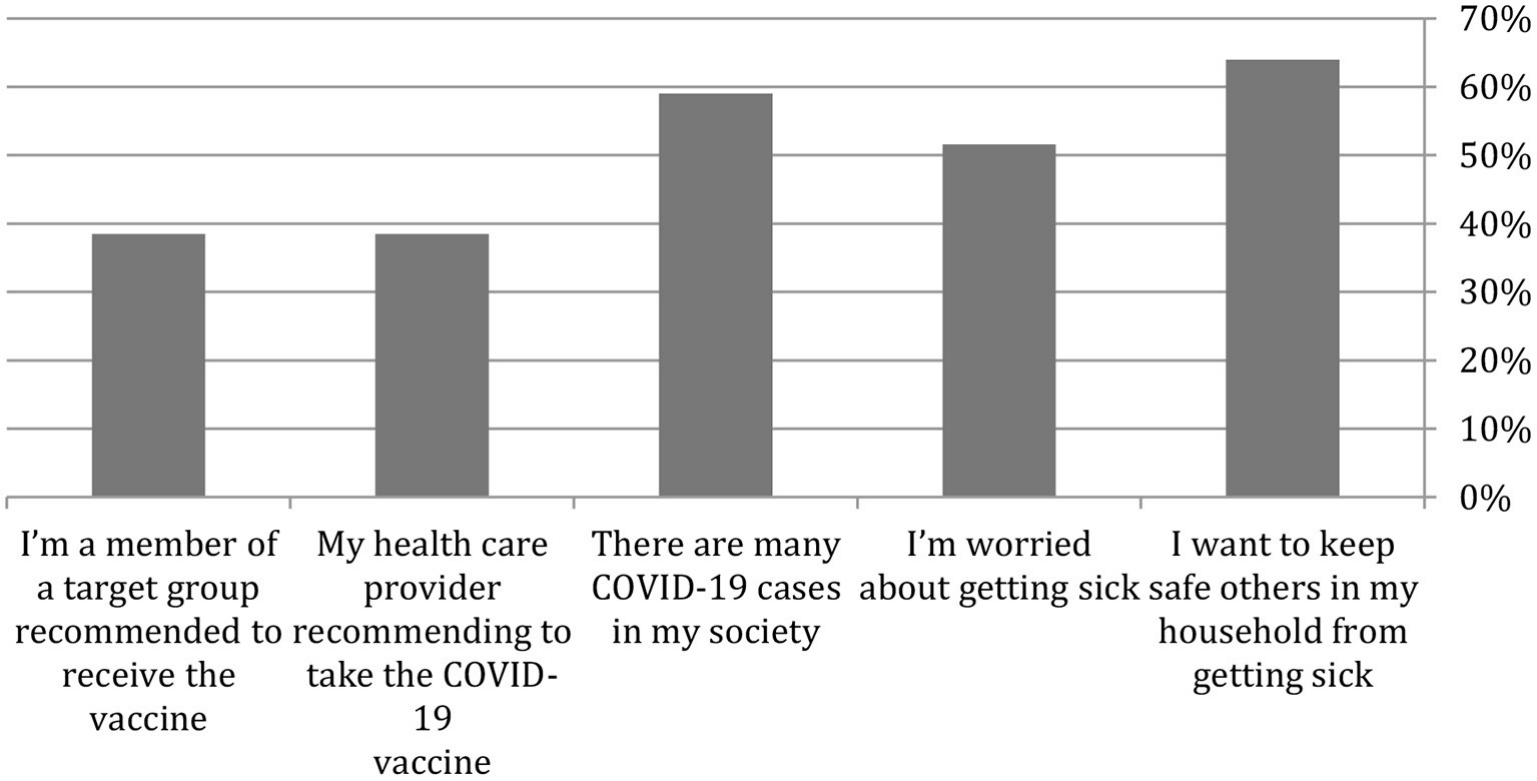
Parental reasons for accepting to vaccinate themselves or one of their family members with a COVID-19 vaccine.

Parents who approved vaccinating their children were asked five questions about the causes for the acceptance. Figure 1 indicates that the vital, compelling reason parents who accepted vaccinating their children with the COVID-19 vaccine were to protect other household members (64%). The second reason was multiple COVID-19 cases in the community (95%).

**Figure 2:**
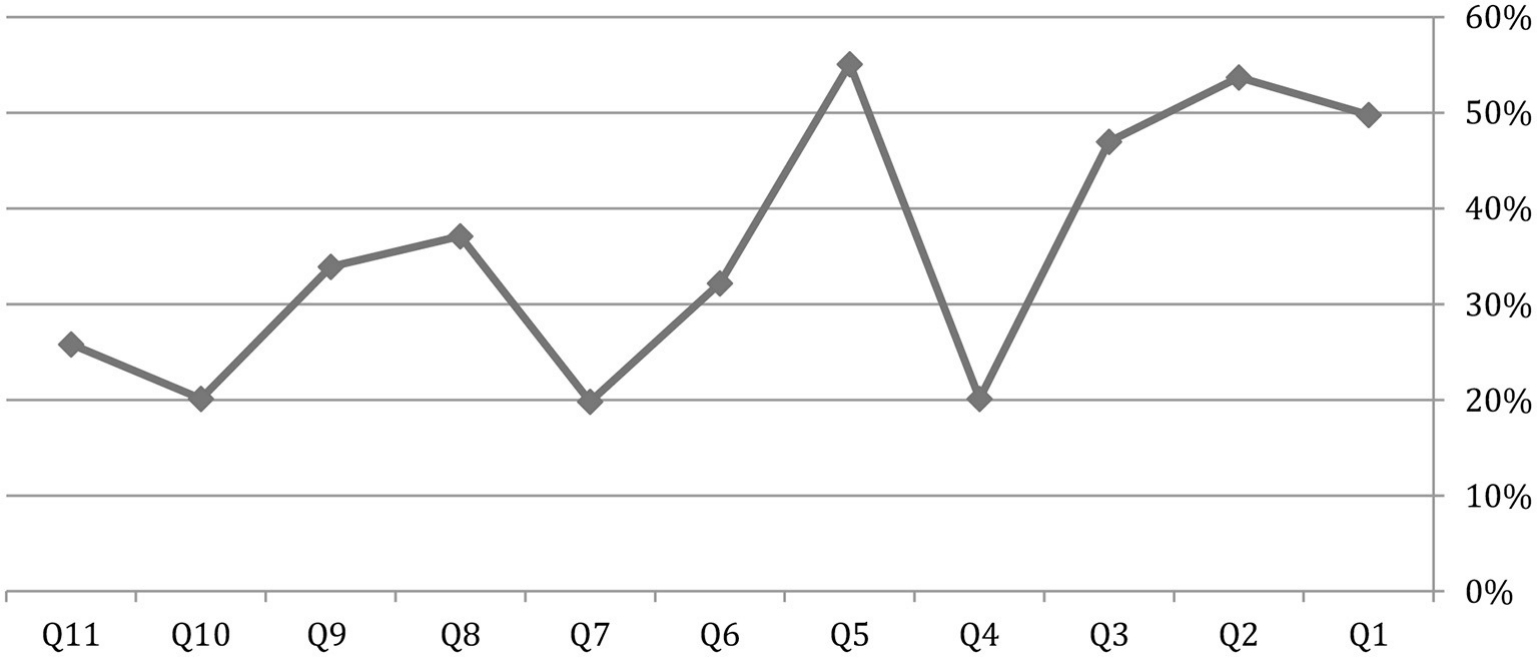
Parental reasons for not accepting to vaccinate themselves or one of their family members with a COVID-19 vaccine.

Parents who refused to vaccinate their children were asked 11 questions (Table 5) about why they refused the vaccine. The main reason the parents refuse to vaccinate their children is that they do not think the vaccine will prevent the infection (55.10%), and the second reason is that they do not know the effectiveness of the vaccine (53.70%) (Figure 2; Table 5).

**Table 5:** Quotes from those not willing to participate in a COVID-19 vaccination campaign.

I'm worried about its side-effects	Q1
Its effectiveness is not known	Q2
The vaccine is too new	Q3
I avoid most vaccines	Q4
I do not think the vaccine will prevent infection	Q5
It's inconvenient to take a vaccine that requires multiple doses	Q6
I do not think COVID-19 virus will cause serious illness even…	Q7
I'm not a member of a target group to receive the vaccine…	Q8
I do not think I will be infected with COVID-19 virus	Q9
I do not have health insurance	Q10
I have immunity because I was already infected with…	Q11

**Figure 3:**
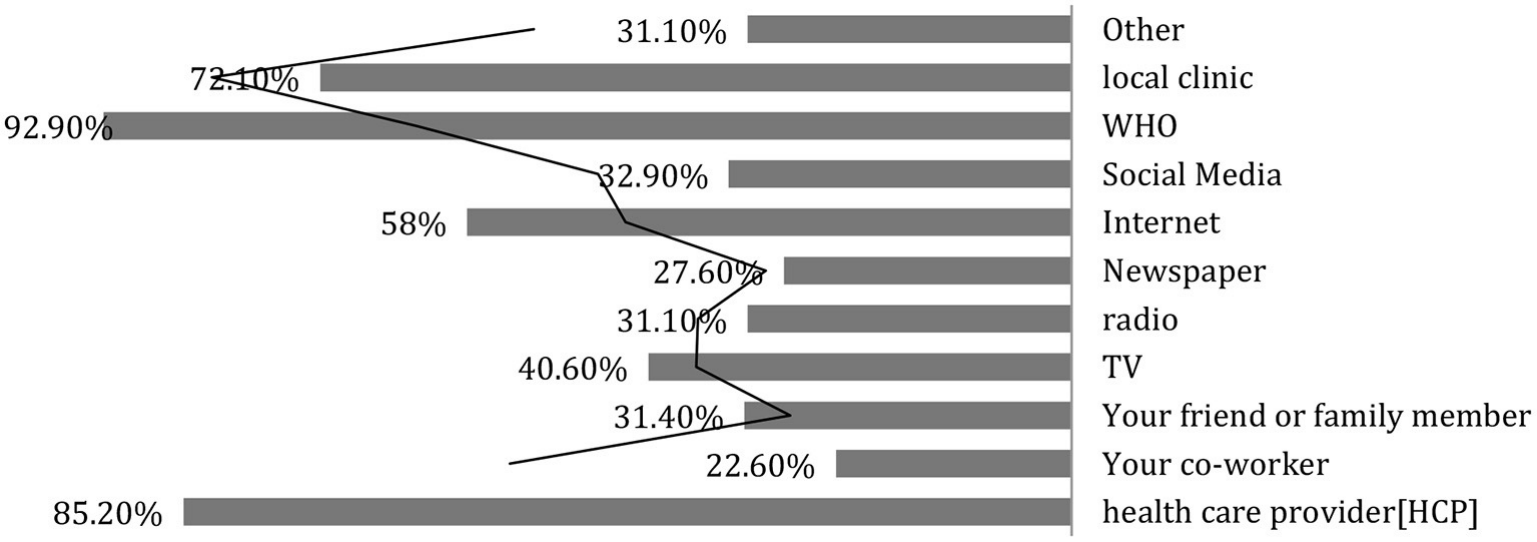
Where would the parents like to get information about the COVID19 vaccine?

Parents were asked about what sources they used to get the information about the COVID-19 vaccine. The WHO website (92.90%) and healthcare provider (85.20%) were the two essential resources of information (Figure 3).

## Discussion

Preventing the spread of COVID-19 requires a joint effort from the public and government. A High vaccination rate in the population could reduce the medical burden, especially in low-income countries where the healthcare systems are more fragile (13). However, we found that only about 37% of parents were willing to vaccinate their children in this study, which was remarkably lower than in other countries (14). It is also worth noticing that over half of the participants questioned the effectiveness of the COVID-19 vaccine. Our findings indicate a need to strengthen public education and identify significant barriers hindering the vaccination of children in this population.

In this study, some specific parental attitudes were associated with the COVID-19 vaccination of their children, including the willingness to vaccinate themselves and participate in clinical trials, trust in the healthcare system and confidence in the domestic vaccine, and concerns about being infected. The body of evidence supports that vaccine hesitance is determined by several intra-personal factors, such as the perceived adverse effects of the vaccine (15, 16). and the perceived severity of the disease (17). External influence, such as the recommendation from healthcare professionals, (18), which is one of the major sources of information on the COVID-19 vaccine found in our study, can also influence parental decision on whether to vaccinate their children or not. Previous studies also found that people prefer domestic over foreign vaccines due to prejudice against foreign countries (19). Our study did not find any association between vaccination of children and the socio-demographic characteristics of the parents, including gender, education, socio-economic and marital status. The findings are consistent with previous studies (20, 21). However, we found that younger parents (18–30 years old) were less likely to vaccinate their children than the older parents (>40 years old), and parents aged 31–40 years were more likely to vaccinate their children than the older parents (>40 years old). This can be possible due o the fact that younger parents were more exposed to social media (22) that disseminates diversified information, including misinformation, that may lead to vaccine hesitancy due to perceived adverse effects of the vaccine (23, 24). Exposure to media could also have, however, positive effects on vaccination. For example, Rubin et al. (25) found that exposure to media was associated with positive health-related behaviors, such as accessing health services, and reducing the use of public transport during flu seasons. Over half of the participants in this study received information about the COVID-19 vaccine from the internet, showing that social media could be a key influence in determining health behaviors. The reason that parents aged 31–40 years were more likely to vaccinate their children than the older parents (>40 years old) in this study is unclear and further investigations are warranted to examine some age-specific beliefs and attitudes toward vaccination. Previous experience in vaccination could also determine the willingness to take the vaccination at present (21). In our study, only 14.8% of participants who received the seasonal flu vaccine were unwilling to vaccinate their children, while over 48% of participants refused to vaccinate their children and themselves. Expectedly, parents who had doubts about the safety and efficacy of the vaccine were associated with not having their children vaccinated which was consistent with previous studies (21, 24, 26).

Based on our findings, several areas a need to be strengthened to improve vaccination rate among children, including public education on the effectiveness of vaccine and confidence on vaccine developed domestically. Health providers have an important role in improving the public trust into the validation of the vaccination process. Thus, better training of health providers could possibly positively impact -eventually- the acceptance of vaccination among the public and could act as key components to improve the public trust to scientific and epidemiological evidence (27).

There is also need to identify age-specific beliefs and attitudes toward vaccination and implement interventions to minimize vaccination barriers among children, especially for young parents aged 18–30 years. As social media is the major source of information for this group of parents, providing the correct information, identifying misinformation and providing clarification by the government officials is of paramount importance to enhance vaccination. There are several reasons why parents are reluctant to vaccinate their children, including the absence of recommendations from primary care pediatricians/physicians, and these results indicate the importance of HCWs as a trusted source in the decision-making process to accept immunizations for COVID-19, as well as the attitudinal nature of both positive factors and obstacles to readiness to vaccinate children. These findings expand our understanding of the intervention targets that will best help youngsters accept receiving the COVID-19 vaccine. COVID-19 vaccinations benefit and risk information should be communicated openly and clearly, ideally *via* health care providers (HCWs). This should be the foundation of interventions aimed at mitigating risk factors and encouraging favorable attitudes toward the vaccine. Vaccination interventions in various settings and populations would have benefitted from the inclusion of enabling variables rather than predisposing factors, as has been proved in the case of HCWs' readiness to undergo influenza vaccination in the context of the COVID-19 pandemic or all other HCWs recommended immunizations, but this was not predetermined.

### The limitations

The cross-sectional has limited the number of participants since it was an online survey due to the internet service in all places, and the internet outage in other places; Thus, many people could not participate. There were no statistics reports about the number of parents in Syria. Reporting a bias in studies is essential; however, in this study, we excluded sampling bias, response bias, nonresponse bias, acquiescence bias, and order bias by writing questions in a clear manner that does not show a bias for one response over the other, and by offering an apparent welcome and background without telling the participants about what's coming up as questions. We did not include the age of the children as a demographic variable in the employed tool since we were imitating a published research in Saudi Arabia; instead, we included the parents of children under the age of 18. Due of the study's small sample size, parents who did not have internet access on their mobile phones or did not have phones at all were unable to participate. Since children and teenagers had not yet participated in the vaccination campaign at the time of the survey, the response that they would accept the COVID-19 vaccine may not accurately reflect actual uptake rates. Additionally, vaccine acceptance levels may fluctuate over time, particularly when vaccine trials and vaccine education campaigns progress.

## Conclusion

Based on our findings, we observed a much lower rate of parent's willingness to vaccinate their children. Lack of knowledge and awareness about the COVID-19 vaccine might be the potential reasons behind this low willingness. However, further studies should investigate the factors associated with hesitancy toward vaccination of children in the Syrian community. This problem plays a major role in the continued spread of the virus among children and its transmission to adults, which may cause an exacerbation of the number of infections and deaths caused by the virus. This constitutes a challenge for the health sector in Syria, which suffers from the lack of equipment and sufficient resources to manage critical cases of COVID-19 patients; thus, more awareness programs should be conducted about the safety, importance, and benefit of the vaccine which is considered to be the only hope in reducing severe cases. In addition, strict laws must be imposed in order to receive the vaccine from government hospitals.

## Data availability statement

The raw data supporting the conclusions of this article will be made available by the authors, without undue reservation.

## Data collection group

Hasan Ibraheem (Faculty of Dentistry, Damascus University; hasanibraheem317@gmail.com)

ZEINA ALEBRAHEM (Faculty of pharmacy, Tishreen University; zeina192019mb@gmail.com)

Rawad Saker (Faculty of pharmacy, Tarous University; Rawadsaker172@gmail.com)

## Ethics statement

The studies involving human participants were reviewed and approved by the Ethics Committee at Aleppo and Damascus University. The participants provided their written informed consent to participate in this study.

## Author contributions

SS: conceptualization, methodology, formal analysis, writing-original draft, review and editing. All authors contributed to the article, writing-review and editing, and approved the submitted version.

## Conflict of interest

The authors declare that the research was conducted in the absence of any commercial or financial relationships that could be construed as a potential conflict of interest.

## Publisher's note

All claims expressed in this article are solely those of the authors and do not necessarily represent those of their affiliated organizations, or those of the publisher, the editors and the reviewers. Any product that may be evaluated in this article, or claim that may be made by its manufacturer, is not guaranteed or endorsed by the publisher.

## References

[1] WHO. WHO Coronavirus (COVID-19) Dashboard. (2022). Available online at: https://covid19.who.int/ (accessed March 02, 2022).

[2] Hodgson SH Mansatta K Mallett G Harris V Emary KR. Pollard AJJTlid. What defines an efficacious COVID-19 vaccine? A review of the challenges assessing the clinical efficacy of vaccines against SARS-CoV-2. Lancet Infect Dis. (2021) 21:e26–35. 10.1016/S1473-3099(20)30773-8 33125914 PMC7837315

[3] Yu MA Shen AK Ryan MJ Boulanger LL. Coordinating COVID-19 vaccine deployment through the WHO COVID-19 Partners Platform. Bull World Health Org. (2021) 99:171–171a. 10.2471/BLT.21.285550 33716336 PMC7941099

[4] WHO. 10 threats to global health. Available online at: https://www.who.int/news-room/spotlight/ten-threats-to-global-health-in-2019 (accessed March 02, 2022).

[5] OCHA. Syrian Arab Republic: 2021 Needs and Response Summary. (2021). Available online at: https://reliefweb.int/report/syrian-arab-republic/syrian-arab-republic-2021-needs-and-response-summary-february-2021 (accessed March 02, 2022).

[6] Frederiksen LSF Zhang Y Foged C Thakur A. The long road toward COVID-19 herd immunity: vaccine platform technologies and mass immunization strategies. Front Immunol. (2020) 11:1817. 10.3389/fimmu.2020.01817 32793245 PMC7385234

[7] Lazarus JV Ratzan SC Palayew A Gostin LO Larson HJ Rabin K. Global survey of potential acceptance of a COVID-19 vaccine. Nat Med. (2021) 27:225–8. 10.1038/s41591-020-1124-9 33082575 PMC7573523

[8] Wong MC Wong EL Huang J Cheung AW Law K Chong MK. Acceptance of the COVID-19 vaccine based on the health belief model: a population-based survey in Hong Kong. Vaccine. (2021) 39:1148–56. 10.1016/j.vaccine.2020.12.083 33461834 PMC7832076

[9] Biasio LR. Immunotherapeutics. Vaccine hesitancy and health literacy. Hum Vaccin Immunother. (2017) 13:701–2. 10.1080/21645515.2016.1243633 27808587 PMC5360145

[10] Lucia VC Kelekar A. Afonso NMJJoPH. COVID-19 vaccine hesitancy among medical students. J Public Health. (2021) 43:445–9. 10.1093/pubmed/fdaa230 33367857 PMC7799040

[11] Shibani M Alzabibi MA Mouhandes AE-F Alsuliman T Mouki A Ismail H. COVID-19 vaccination acceptance among Syrian population: a nationwide cross-sectional study. BMC Public Health. (2021) 21:1–12. 10.1186/s12889-021-12186-6 34789229 PMC8598277

[12] Ennaceur S Al-Mohaithef M. Parents' willingness to vaccinate children against COVID-19 in Saudi Arabia: a cross-sectional study. Vaccines. (2022) 10:156. 10.3390/vaccines10020156 35214616 PMC8875640

[13] Wagner CE Saad-Roy CM Grenfell BTJNRI. Modeling vaccination strategies for COVID-19. Nat Rev Immunol. (2022) 22:139–41. 10.1038/s41577-022-00687-3 35145245 PMC8830981

[14] Bell S Clarke R Mounier-Jack S Walker JL. Paterson PJV. Parents' and guardians' views on the acceptability of a future COVID-19 vaccine: A multi-methods study in England. Vaccine. (2020) 38:7789–98. 10.1016/j.vaccine.2020.10.027 33109389 PMC7569401

[15] Quinn SC Kumar S Freimuth VS Kidwell K Musa D. Public willingness to take a vaccine or drug under Emergency Use authorization during the 2009 H1N1 pandemic. Biosecur Bioterror. (2009) 7:275–90. 10.1089/bsp.2009.0041 19775200 PMC2998968

[16] Lau JT Yeung NC Choi KC Cheng MY Tsui HY Griffiths S. Factors in association with acceptability of A/H1N1 vaccination during the influenza A/H1N1 pandemic phase in the Hong Kong general population. Vaccine. (2010) 28:4632–7. 10.1016/j.vaccine.2010.04.076 20457289 PMC7131323

[17] Maurer J Uscher-Pines L. Harris KM. Perceived seriousness of seasonal and A(H1N1) influenzas, attitudes toward vaccination, and vaccine uptake among US adults: does the source of information matter? Prev Med. (2010) 51:185–7. 10.1016/j.ypmed.2010.05.008 20510270

[18] Schwarzinger M Flicoteaux R Cortarenoda S Obadia Y Moatti JP. Low acceptability of A/H1N1 pandemic vaccination in French adult population: did public health policy fuel public dissonance? PLoS ONE. (2010) 5:e10199. 10.1371/journal.pone.0010199 20421908 PMC2856629

[19] Kobayashi Y Howell C Heinrich T. Vaccine hesitancy, state bias, and COVID-19: evidence from a survey experiment using Phase-3 results announcement by BioNTech and Pfizer. Soc Sci Med. (2021) 282:114115. 10.1016/j.socscimed.2021.114115 34157613 PMC8205290

[20] Nguyen T Henningsen KH Brehaut JC Hoe E. Wilson K. Acceptance of a pandemic influenza vaccine: A systematic review of surveys of the general public. Infect Drug Resist. (2011) 4:97–207. 10.2147/IDR.S23174 22114512 PMC3215344

[21] Horney JA Moore Z Davis M. MacDonald PD. Intent to receive pandemic influenza A (H1N1) vaccine, compliance with social distancing and sources of information in NC. PLoS ONE. (2009) 5:e11226. 10.1371/journal.pone.0011226 20585462 PMC2887902

[22] Bartholomew MK Schoppe-Sullivan SJ Glassman M Kamp Dush CM Sullivan JM. New parents' Facebook use at the transition to parenthood. Fam Relat. (2012) 61:455–69. 10.1111/j.1741-3729.2012.00708.x 23671354 PMC3650729

[23] Wilson SL Wiysonge CJBGH. Social media and vaccine hesitancy. BMJ Glob Health. (2020) 5:e004206. 10.1136/bmjgh-2020-004206 33097547 PMC7590343

[24] Raut S Apte A Srinivasan M Dudeja N Dayma G Sinha B. Determinants of maternal influenza vaccination in the context of low- and middle-income countries: a systematic review. PLoS ONE. (2022) 17:e0262871. 10.1371/journal.pone.0262871 35081138 PMC8791521

[25] Rubin GJ Potts HW Michie S. The impact of communications about swine flu (influenza A H1N1v) on public responses to the outbreak: results from 36 national telephone surveys in the UK. Health Technol Assess. (2010) 14:183–266. 10.3310/hta14340-03 20630124

[26] Kricorian K Civen R Equils OJHV. Immunotherapeutics, COVID-19 vaccine hesitancy: misinformation and perceptions of vaccine safety. Hum Vaccines Immunother. (2022) 18:1950504. 10.1080/21645515.2021.1950504 34325612 PMC8920251

[27] Bianco A Mascaro V Zucco R Pavia M. Parent perspectives on childhood vaccination: how to deal with vaccine hesitancy and refusal? Vaccine. (2019) 37:984–90. 10.1016/j.vaccine.2018.12.062 30655175

