# Supplementary material for: Parents' acceptance to vaccinate children against COVID-19: A Syrian online survey
Source: Front Public Health. 2022 Oct 13;10:955362. doi: 10.3389/fpubh.2022.955362 (PMC9610112; doi:10.3389/fpubh.2022.955362)
Supplement: Supplementary file 1 [file Table_1.DOCX]

|  | Baseline charactaristic | | | |
| --- | --- | --- | --- | --- |
| 1 | Age (years) |  | | |
|  | | | | |
| 2 | Gender | Female | 1 |  |
|  |  | Male | 2 |  |
|  | | | | |
| 3 | Marital Status | Married | 1 |  |
|  |  | Single | 2 |  |
|  |  | Separated | 3 |  |
|  |  | Divorced | 4 |  |
|  |  | Widowed | 5 |  |
|  | | | | |
| 4 | Education | Postgraduate | 1 |  |
|  |  | Graduate | 2 |  |
|  |  | High School | 3 |  |
|  |  | Primary School | 4 |  |
|  |  | No Education | 5 |  |
|  | | | | |
| 5 | Occupation | Govt. | 1 |  |
|  |  | Private | 2 |  |
|  |  | Self-employed | 3 |  |
|  |  | Not working | 4 |  |
|  | | | | |
| 6 | What is your socio-economic status in the society | Low | 1 |  |
|  |  | Medium | 2 |  |
|  |  | High | 3 |  |
|  | | | | |
| 7 | Family income |  | | |
|  | | | | |
| 8 | How many members in your family | 1 | 1 |  |
|  |  | 2 | 2 |  |
|  |  | 3 | 3 |  |
|  |  | 4 | 4 |  |
|  |  | 5 | 5 |  |
|  |  | >5 | 6 |  |
|  | | | | |
| Parents’ Willingness to Vaccinate Children against COVID-19 | | | | |
|  | Consent | Yes | 1 |  |
|  |  | No | 2 |  |
|  | | | | |
| 1 | Mobile or Contact number |  | | |
|  | | | | |
| 2 | Are you working in a healthcare system? | Yes | 1 |  |
|  |  | No | 2 |  |
|  | | | | |
| 3 | Place of work | Government Hospital | 1 |  |
|  |  | Private Hospital | 2 |  |
|  | | | | |
| 4 | Type of health care personnel | Medical doctor | 1 |  |
|  |  | Physician assistant | 2 |  |
|  |  | Registered nurse | 3 |  |
|  |  | Assistant nurse, nurse technician | 4 |  |
|  |  | Radiology/ X-ray technician | 5 |  |
|  |  | Phlebotomist | 6 |  |
|  |  | Opthalmologist | 7 |  |
|  |  | Physical therapist | 8 |  |
|  |  | Nutritionist/ dietician | 9 |  |
|  |  | Midwife | 10 |  |
|  |  | Pharmacist | 11 |  |
|  |  | Pharmacy technician/ dispenser | 12 |  |
|  |  | Lab personnel | 13 |  |
|  |  | Admission/ reception clerk | 14 |  |
|  |  | Patient transporter | 15 |  |
|  |  | Caterng staff | 16 |  |
|  |  | Cleaner | 17 |  |
|  |  | MSW | 18 |  |
|  |  | Other | 19 |  |
|  | | | | |
| 5 | Do you have a history of staying in the same  household or environment with a confirmed COVID-  19 patient? | Yes | 1 |  |
|  |  | No | 2 |  |
|  |  | Don't Know | 3 |  |
|  | | | | |
| 6 | Do you have history of traveling together in close  proximity (within 1 meter) with a confirmed COVID-  19 patient in any kind of conveyance? | Yes | 1 |  |
|  |  | No | 2 |  |
|  |  | Don't Know | 3 |  |
|  | | | | |
| 7 | How concerned are you that you or someone in your  family will be infected with COVID-19virus? | Yes | 1 |  |
|  |  | No | 2 |  |
|  |  | Don't Know | 3 |  |
|  | | | | |
| 8 | Do you have trust in the healthcare system to  manage the current situation related to COVID-19? | Yes | 1 |  |
|  |  | No | 2 |  |
|  |  | Don't Know | 3 |  |
|  | | | | |
| 9 | Did you receive a *seasonal* flu vaccine for the 2019-  2020 flu season (last year)? | Yes | 1 |  |
|  |  | No | 2 |  |
|  |  | Don't Know | 3 |  |
|  | | | | |
| 10 | Have you ever received a seasonal flu vaccine? | Yes | 1 |  |
|  |  | No | 2 |  |
|  |  | Don't Know | 3 |  |
|  | | | | |
| 11 | Have you ever refused a vaccine for yourself or a child because you considered it as useless or dangerous? | Yes | 1 |  |
|  |  | No | 2 |  |
|  |  | Don't Know | 3 |  |
|  | | | | |
| 12 | Have you ever postponed a vaccine recommended by a physician? | Yes | 1 |  |
|  |  | No | 2 |  |
|  |  | Don't Know | 3 |  |
|  | | | | |
| 13 | Have you ever had a vaccine for a child or yourself despite doubts about its efficacy? | Yes | 1 |  |
|  |  | No | 2 |  |
|  |  | Don't Know | 3 |  |
|  | | | | |
| 14 | Before this interview, were you aware that COVID- 19virus is currently circulating in the community? | Yes | 1 |  |
|  |  | No | 2 |  |
|  |  | Don't Know | 3 |  |
|  | | | | |
| 15 | To the best of your knowledge, is there currently a vaccine being prepared for the pandemic Coronavirus strain referred to as COVID-19 vaccine? | Yes | 1 |  |
|  |  | No | 2 |  |
|  |  | Don't Know | 3 |  |
|  |  |  |  |  |
|  | | | | |
| 16 | Do you intend to participate in a COVID-19 vaccine clinical trial | Yes | 1 |  |
|  |  | No | 2 |  |
|  |  | Don't Know | 3 |  |
|  | | | | |
| 17 | Do you intend to get vaccinated against Coronavirus when the vaccine is available? | Yes | 1 |  |
|  |  | No | 2 |  |
|  |  | Don't Know | 3 |  |
|  | | | | |
| 18 | If NO:I’m going to read a list of possible reasons why someone might choose not to get the COVID-19 vaccine. Please tell me if each of the following is a reason that you do not intend to get vaccinated. | | | |
| a. | The vaccine Is too new | Yes  No | 1  2 |  |
| b. | Its effectiveness is not known | Yes  No | 1  2 |  |
| c. | I’m worried about its | Yes  No | 1  2 |  |
| d. | I avoid most vaccines | Yes  No | 1  2 |  |
| e. | I do not think I will be infected with COVID- | Yes  No | 1  2 |  |
| f. | I do not think COVID-19will cause serious illness  even if I am infected | Yes  No | 1  2 |  |
| g. | I do not think the vaccine will prevent infection | Yes  No | 1  2 |  |
| h. | It’s inconvenient to take a vaccine that requires  several doses | Yes  No | 1  2 |  |
| i. | I’m worried about possible side effects of COVID-19 | Yes  No | 1  2 |  |
| j. | I’m not a member of a target group to receive the  vaccine, such as a health care worker, adult above aged50, or a pregnant woman | Yes No | 1  2 |  |
| k. | My religion prevents vaccination | Yes  No | 1  2 |  |
| l. | It might cost too much | Yes  No | 1  2 |  |
| m. | I do not know where to get vaccinated | Yes  No | 1  2 |  |
| n. | I do not have transportation to get vaccinated | Yes  No | 1  2 |  |
| o. | I have immunity because I was already infected with COVID-19 | Yes No | 1  2 |  |
| p. | I do not have health insurance | Yes  No | 1  2 |  |
| q. | Other | Yes  No | 1  2 |  |
|  | | | | |
| 19 | If YES:Please tell me if each of the following is a  reason that you intend to get vaccinated against COVID-19. Y/N for each option below. |  |  |  |
| a. | I’m worried about getting sick | Yes  No | 1  2 |  |
| b. | There are many COVID cases in my society | Yes  No | 1  2 |  |
| c. | I’m a member of a target group recommended to  receive the vaccine, such as a health care worker, adult above aged50, or a pregnant woman. | Yes No | 1  2 |  |
| d. | My health care provider recommending to take the  COVID-19 vaccine | Yes  No | 1  2 |  |
| e. | I want to keep others in my household from getting  sick | Yes  No | 1  2 |  |
| f. | Other |  |  |  |
|  | | | | |
| 20 | If not sure:Please tell me if you have following  concerns about the vaccine. |  |  |  |
| a | The vaccine is too new | Yes  No | 1  2 |  |
| b. | Its effectiveness is not known | Yes  No | 1  2 |  |
| c | Its side effect is not known | Yes  No | 1  2 |  |
| d | Its doses are not known | Yes  No | 1  2 |  |
| e. | It might cost too much | Yes  No | 1  2 |  |
| f. | Other | Yes  No | 1  2 |  |
|  | | | | |
| 21 | If Yes: Where do you intend to get the COVID1 vaccine? | Private Health Care Provider  Local Health  Department Hospital  Community health clinic | 1  2  3  4 |  |
| 22 | Do you have any children age 18 or under living at home? | Yes  No | 1  2 |  |
| 23 | Do you intend to vaccinate your child(ren) for COVID- 19? | Yes  No  Don't Know | 1  2  3 |  |
| 24 | What should be the ideal dose of the COVID-19 vaccine | One Two Three Other | 1  2  3  4 |  |
| 25 | Which of the following manufacturer/brand of the vaccine you would like to receive | Domstic Brand Imported Brand  Not Sure | 1  2  3 |  |
| 26 | How confident you are on domestic vaccines | Better than abroad Similar  Worse  Not Sure | 1  2  3  4 |  |
| 27 | What kind of health insurance do you have? | Private Insurance Government Insurance  I don't have Health Insurance Other  Don't Know | 1  2  3  4  5 |  |
| 28 | Does your insurance provider cover your vaccine cost? | Yes  No  Don't Know | 1  2  3 |  |
| 29 | What should be the ideal cost [Rupees] of the vaccine? | Free  100  500  1000  Other | 1  2  3  4  5 |  |
|  | | | | |
| 30 | What percentage of the vaccine cost you plan to pay comfortable? | 0  25  50  75  100 | 1  2  3  4  5 |  |
|  | | | | |
| 31 | Where did you intend to get information about  COVID-19vaccine? |  |  |  |
| a | A health care provider | Yes  No | 1  2 |  |
| b. | Employer or a co-worker | Yes  No | 1  2 |  |
| c | Family member or friend | Yes  No | 1  2 |  |
| d | Television | Yes  No | 1  2 |  |
| e. | radio | Yes  No | 1  2 |  |
| f. | Newspaper | Yes  No | 1  2 |  |
| g. | Internet | Yes  No | 1  2 |  |
| h. | Social Media | Yes  No | 1  2 |  |
| i | Trusted website like Ministry of Health/ WHO/ CDC | Yes  No | 1  2 |  |
| j. | State or Local health Department | Yes  No | 1  2 |  |
| k | Other | Yes  No | 1  2 |  |
|  | | | | |
| 32 | Please rank the following information sources for COVID-19vaccine information?[check from any of the following sources]? |  |  |  |
| a | A health care provider | Most trusted  somehow truste  d not atall trusted | 1  2  3 |  |
| b. | Employer or a co-worker | Most trusted  somehow truste  d not atall trusted | 1  2  3 |  |
| c | Family member or friend | Most trusted  somehow truste  d not atall trusted | 1  2  3 |  |
| d | Television | Most trusted  somehow truste  d not atall trusted | 1  2  3 |  |
| e. | Radio | Most trusted  somehow truste  d not atall trusted | 1  2  3 |  |
| f. | Newspaper | Most trusted  somehow truste  d not atall trusted | 1  2  3 |  |
| g. | Internet | Most trusted  somehow truste  d not atall trusted | 1  2  3 |  |
| h. | Social Media | Most trusted  somehow truste  d not atall trusted | 1  2  3 |  |
| i | Trusted website like Ministry of Health/ WHO/ CDC | Most trusted  somehow truste  d not atall trusted | 1  2  3 |  |
| j. | State or Local health Department | Most trusted  somehow truste  dnot atall trusted | 1  2  3 |  |
